# Early Protraction Facemask Followed by Fixed Appliance Therapy in a Growing Patient With Skeletal Class III Malocclusion: A Case Report

**DOI:** 10.1155/crid/5584944

**Published:** 2026-06-08

**Authors:** Arron Yan Ran Lim, Sock Nee Tey, Khairil Aznan Mohamed Khan

**Affiliations:** ^1^ Orthodontic Unit, Department of Family Oral Health, Faculty of Dentistry, The National University of Malaysia (UKM), Kuala Lumpur, Malaysia, ukm.my; ^2^ Department of Orthodontics, Parit Buntar Dental Clinic, Ministry of Health Malaysia, Parit Buntar, Malaysia, moh.gov.my

**Keywords:** case report, cephalometric analysis, fixed appliance therapy, growth modification, malocclusion, skeletal Class III, protraction facemask

## Abstract

**Background:**

Skeletal Class III malocclusion remains a significant orthodontic challenge because of its multifactorial aetiology, strong genetic influence, and the unpredictability of mandibular growth. Although early protraction facemask therapy is widely advocated for maxillary deficiency, the optimal timing of intervention and long‐term stability remain controversial. This report describes successful management of a growing patient with borderline skeletal Class III malocclusion using a non‐expansion facemask protocol initiated beyond the conventionally recommended early treatment age.

**Case Presentation:**

A 10.5‐year‐old female presented with anterior crossbite, mild skeletal Class III malocclusion, maxillary retrusion and an anterolateral functional mandibular shift, with a positive family history of mandibular prognathism. Orthopaedic treatment was initiated at cervical vertebral maturation stage CS2 using a non‐expansion protraction facemask protocol, followed by comprehensive non‐extraction fixed appliance therapy.

**Intervention and Outcomes:**

Serial clinical and cephalometric evaluations demonstrated forward displacement of the maxilla, improvement in sagittal skeletal relationship, favourable dentoalveolar compensation, elimination of functional mandibular shift and improvement in facial profile and smile aesthetics. At treatment completion, stable Class I canine and molar relationships, positive overjet and overbite, improved facial symmetry and balanced facial proportions were achieved.

**Conclusion:**

This case highlights that biologically timed protraction facemask therapy, even when initiated beyond the conventional early age, combined with careful case selection and comprehensive fixed appliance treatment, may achieve favourable functional and aesthetic outcomes in selected growing patients with borderline skeletal Class III malocclusion.

## 1. Introduction

Skeletal Class III malocclusion is a complex condition arising from interactions between genetic predisposition and environmental factors, resulting in maxillary retrusion, mandibular prognathism or a combination of both. There is also a distinct entity of pseudo‐Class III cases, in which a forward mandibular functional shift masks the true underlying skeletal relationship, and recognition and elimination of the shift are critical to accurate diagnosis and treatment planning. The prevalence of Class III malocclusion is higher in East and Southeast Asian populations, including Malaysia [1–3], making early diagnosis and management clinically relevant. In growing patients with maxillary deficiency, protraction facemask therapy has been advocated to modify skeletal growth and improve the sagittal intermaxillary relationship. Although short‐term benefits are well documented [4–6], treatment outcomes remain influenced by growth pattern, timing of intervention, and patient compliance [7]. There remains limited clinical evidence regarding the effectiveness of facemask therapy initiated above 10 years old, particularly without maxillary expansion and followed by comprehensive fixed appliance therapy in borderline skeletal Class III cases. This report presents a CARE‐compliant case illustrating favourable skeletal, dental and aesthetic outcomes following early protraction facemask therapy initiated at a biologically appropriate growth stage and followed by comprehensive fixed appliance treatment.

## 2. Patient Information

A 10.5‐year‐old female presented for orthodontic assessment with concerns regarding irregular teeth and an anterior crossbite, described by her parent as an ‘underbite’. The patient was medically fit and well, with no history of systemic disease, trauma, oral habits or previous orthodontic treatment. A significant family history of mandibular prognathism was reported on the maternal side, including relatives who had undergone or were undergoing combined orthodontic–orthognathic treatment. No relevant psychosocial issues were identified.

## 3. Clinical Findings

Extraoral examination revealed a concave facial profile with midface retrusion, reduced lower anterior facial height and a low mandibular plane angle. A frontal facial analysis was performed using vertical reference lines and mild lower facial asymmetry with 2 mm chin deviation towards the right side was noted.

The temporomandibular joints were asymptomatic; however, a 2‐mm forward mandibular functional shift was observed from centric relation to maximum intercuspation. The centric relation was recorded using a combined operator‐guided and patient‐guided approach. Light gentle pressure was applied digitally to the patient′s chin point to provide guidance, while the patient was instructed to place the tongue against the palate while closing. The techniques have been recommended in clinical reviews of mandibular guidance techniques [8]. Lip competence was present, with reduced maxillary incisor display on smiling.

**Figure 1:**
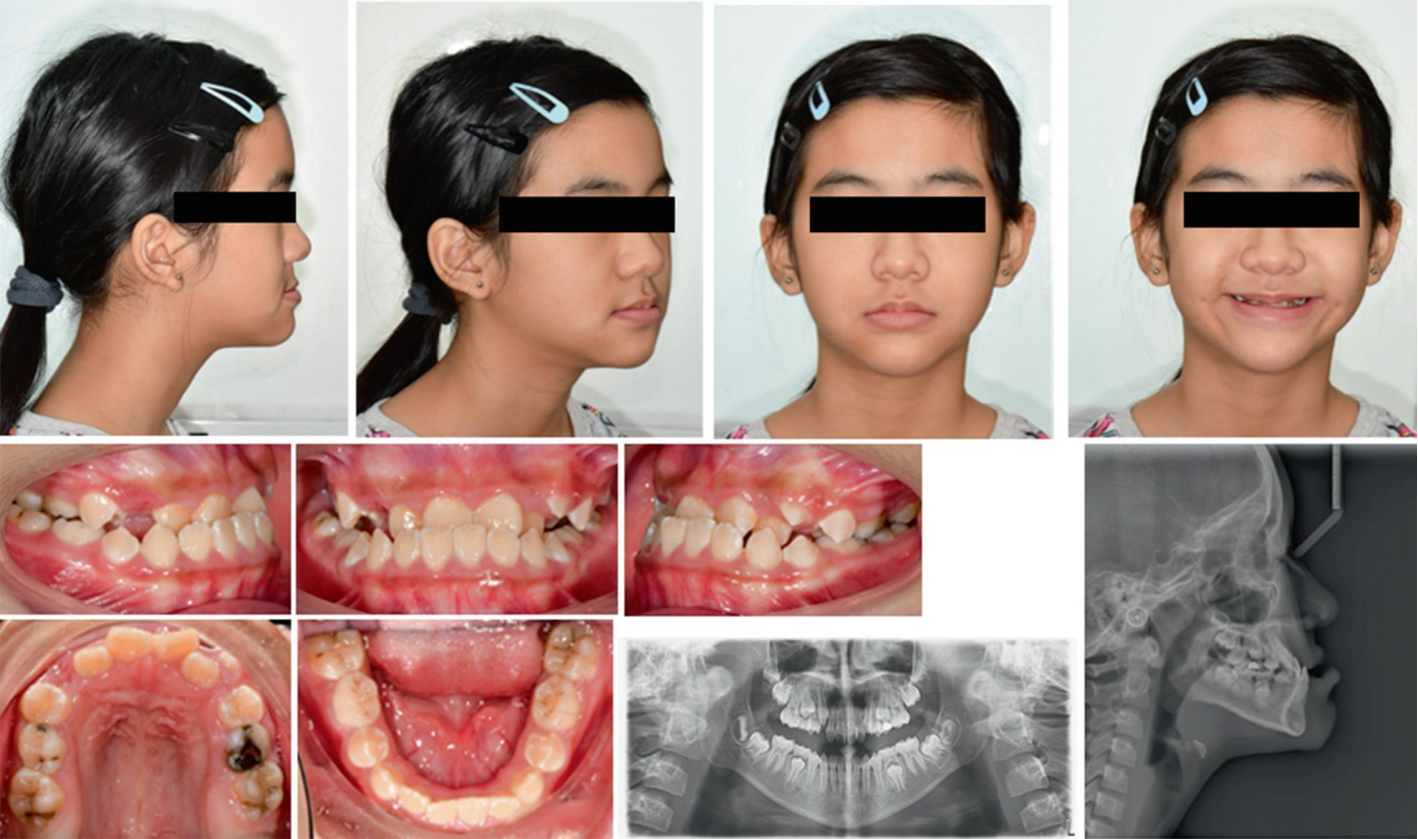
Pretreatment extraoral and intraoral photographs, panoramic radiograph and lateral cephalometric radiograph demonstrating a skeletal Class III pattern with anterior crossbite and reduced incisal display.

Intraoral examination showed late mixed dentition with moderate maxillary crowding (approximately 6 mm) and mild mandibular crowding (approximately 1 mm). The occlusion demonstrated a Class III incisor relationship with a reverse overjet of 2 mm and an increased overbite of approximately 50%. The maxillary dental midline was deviated by 3 mm to the left, whereas the mandibular midline was deviated to the right of facial midline by 2 mm; however, it was coincident with the chin point. There was unilateral posterior crossbite on the right buccal segment, indicating the presence of an anterolateral functional mandibular shift towards the right. A half‐unit Class III molar relationship was present bilaterally (Figure 1).

## 4. Timeline

The timeline of clinical events are as in Table 1

**Table 1:** Timeline of diagnosis, treatment and outcomes.

Age/timepoint	Clinical event
10.5 years	Initial presentation and diagnosis
CS2 stage	Initiation of protraction facemask therapy
+ 4 months	Positive overjet achieved
+ 9 months	Completion of orthopaedic phase
+ 15 months	Initiation of fixed appliance therapy
+ 3.5 years	Completion of treatment and retention

## 5. Diagnostic Assessment

Radiographic examination revealed normal condylar morphology and the presence of all permanent teeth, including third molars. Lateral cephalometric analysis confirmed a skeletal Class III relationship (ANB −4°), with maxillary incisor proclination and mandibular incisor retroclination consistent with dentoalveolar compensation. Cervical vertebral maturation assessment indicated Stage CS2, corresponding to the prepubertal growth phase [9].

The primary diagnosis was skeletal Class III malocclusion with maxillary retrusion and mild mandibular prognathism, complicated by an anterolateral functional shift (Figure 1).

## 6. Therapeutic Intervention

**Figure 2:**
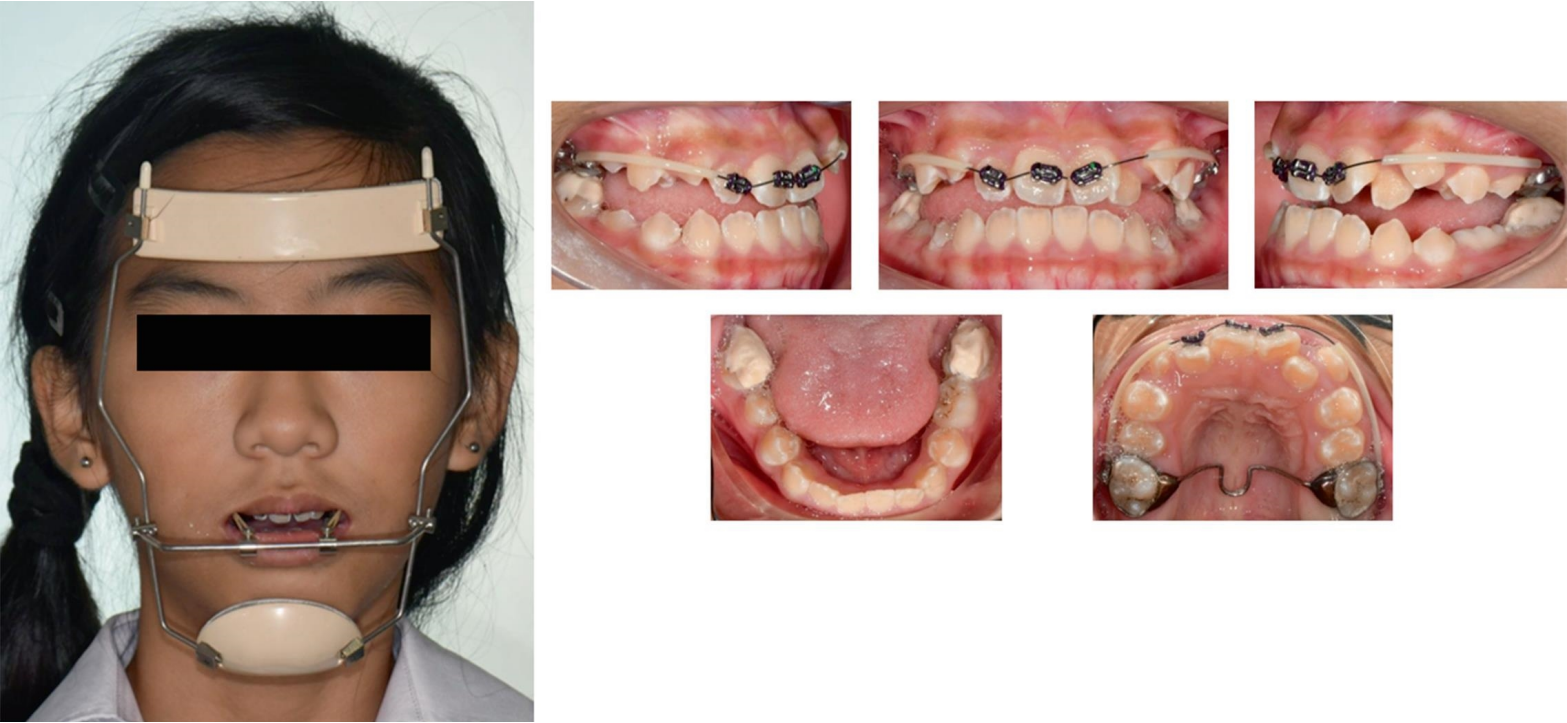
Phase 1 treatment showing protraction facemask therapy combined with a 2 × 4 maxillary fixed appliance and transpalatal arch.

**Figure 3:**
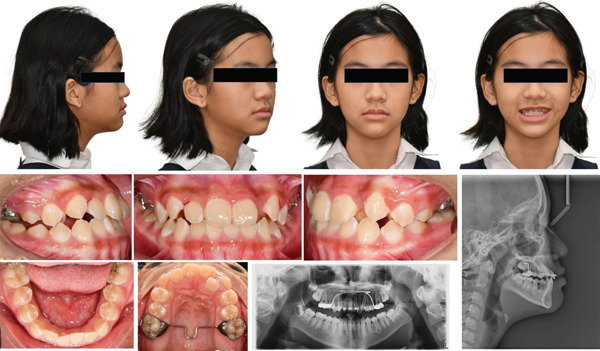
Postorthopaedic extraoral and intraoral photographs, panoramic radiograph and lateral cephalometric radiograph following completion of protraction facemask therapy.

A two‐phase treatment approach was adopted. Phase 1 consisted of orthopaedic protraction facemask therapy using a nonexpansion protocol. A transpalatal arch and a 2 × 4 maxillary fixed appliance were placed, and a Delaire protraction facemask delivering approximately 340 g of force per side was prescribed for 12–14 h daily. Posterior bite blocks were used to facilitate correction of the anterior crossbite (Figures 2 and 3).

**Figure 4:**
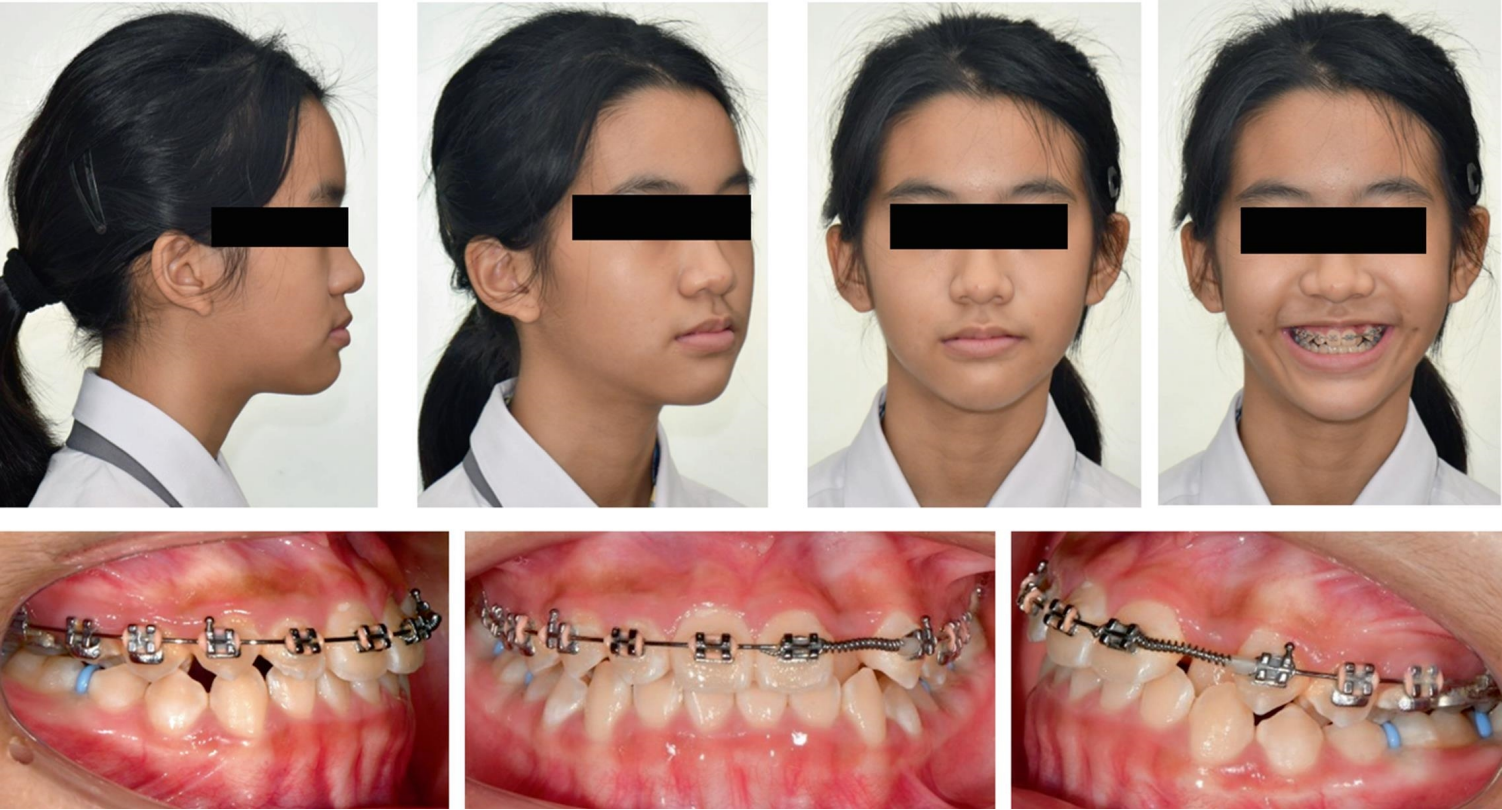
Intraoral photographs 6 months after initiation of Phase 2 fixed appliance therapy, demonstrating maintenance of positive overjet and overbite.

**Figure 5:**
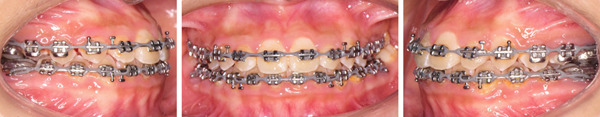
Intraoral photographs after 20 months of comprehensive fixed appliance therapy showing near‐complete alignment and occlusal detailing.

Phase 2 involved comprehensive nonextraction fixed appliance therapy following stabilization of the orthopaedic correction (Figure 4). Conventional archwire sequencing and light interarch elastics were used for alignment, space management and finishing. Retention was provided with removable Hawley retainers (Figure 5).

## 7. Follow‐Up and Outcomes

**Figure 6:**
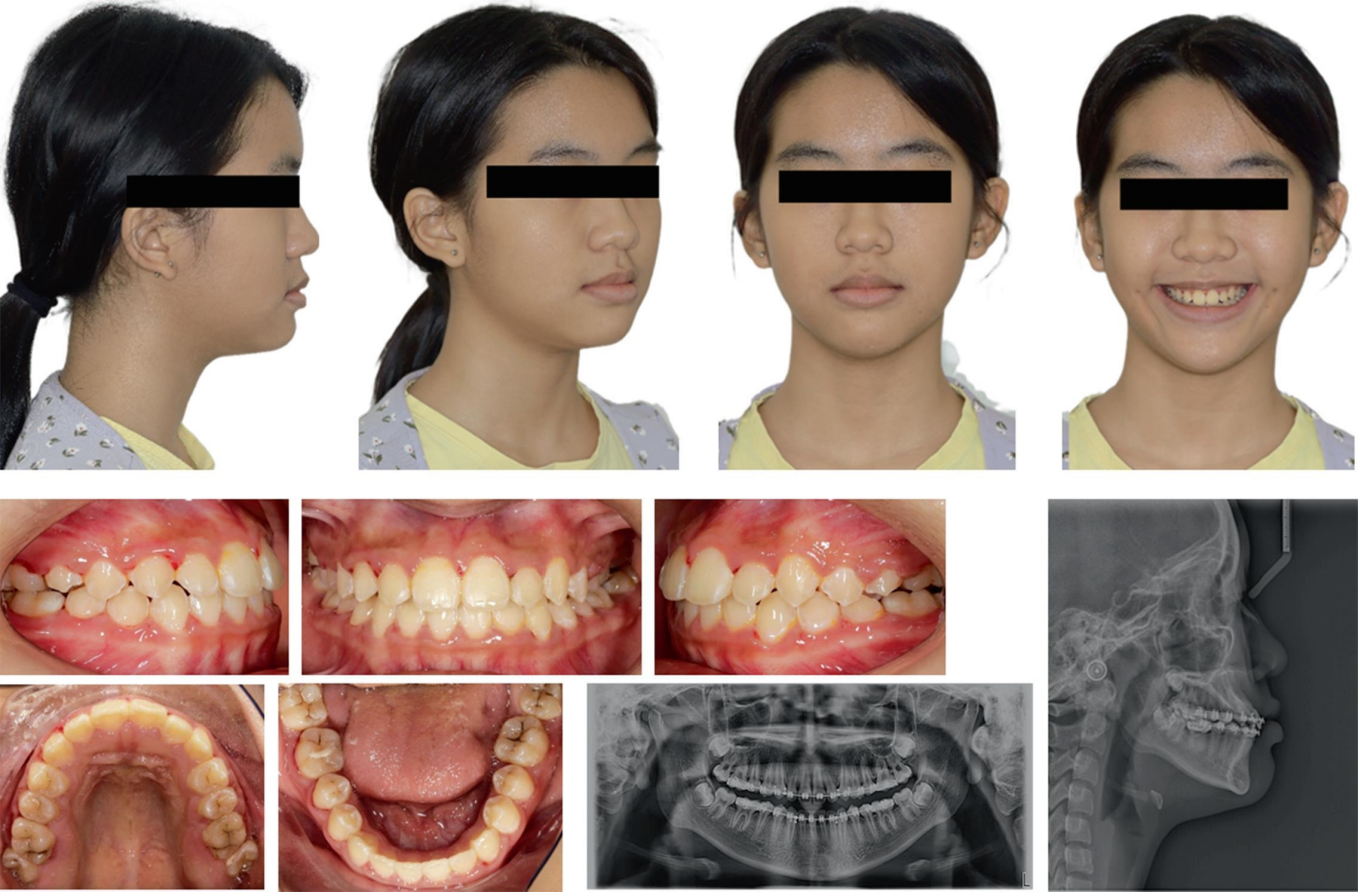
Posttreatment extraoral and intraoral photographs with panoramic and lateral cephalometric radiographs illustrating final occlusal and facial outcomes.

**Figure 7:**
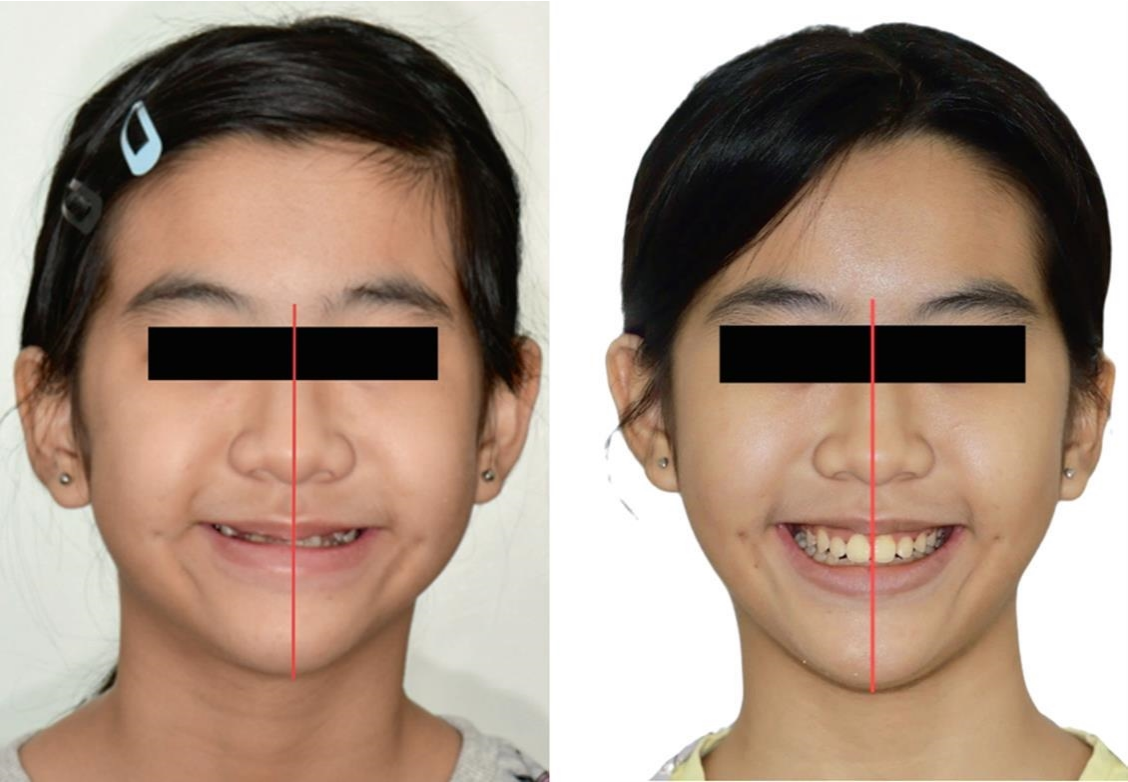
Extraoral facial analysis of pretreatment extraoral photographs compared with posttreatment, demonstrating coinciding facial and dental midlines after eliminating the pretreatment anterolateral mandibular functional shift.

**Figure 8:**
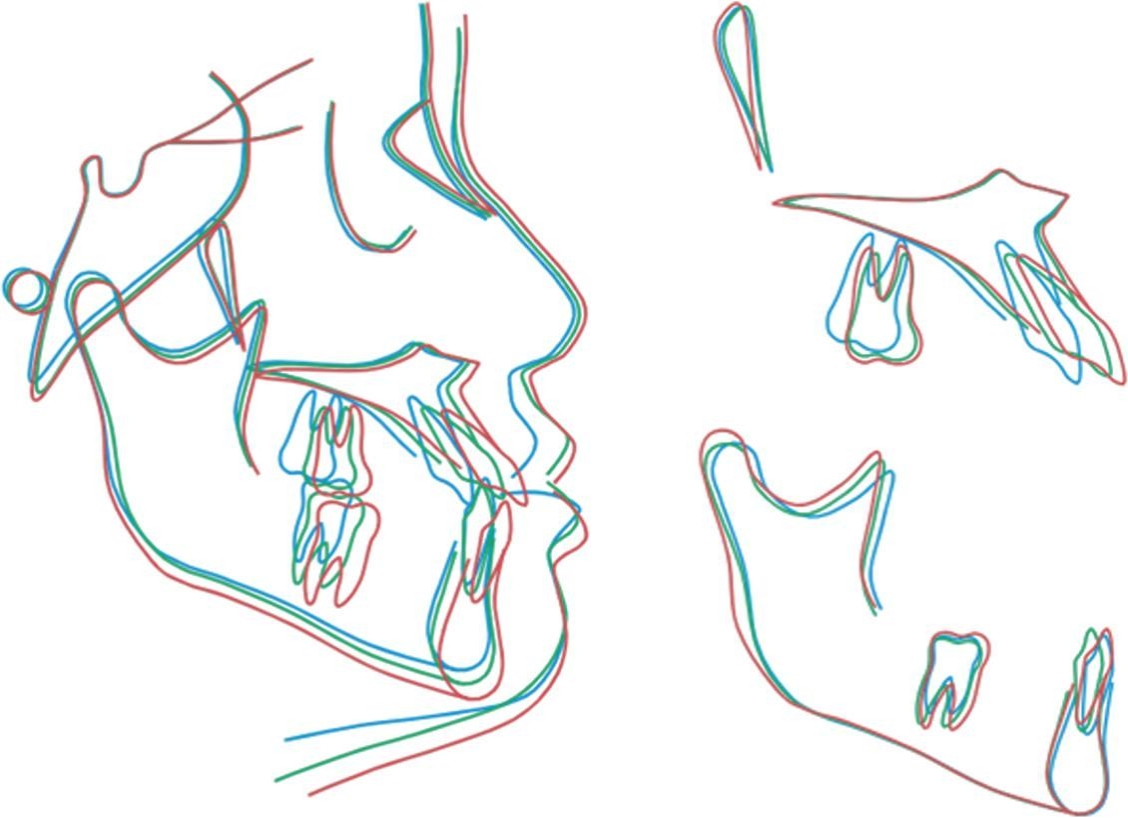
Superimposition of serial lateral cephalometric tracings. Blue lines represent pretreatment, green lines represent postorthopaedic, and red lines represent posttreatment tracings.

After approximately 3.5 years of treatment, the patient exhibited a straight facial profile, improved smile aesthetics, competent lips and balanced vertical facial proportions. Stable Class I canine and molar relationships were achieved, with a positive overjet of 2 mm and an overbite of approximately 30% (Figure 6). Besides, improvement in facial symmetry was observed, consistent with elimination of the mandibular functional displacement. Improvement in lateral head and neck posture was also observed posttreatment, likely reflecting adaptation to the corrected maxillo‐mandibular transverse relationship and improved occlusal stability (Figure 7). Cephalometric analysis in Table 2 demonstrated forward displacement of the maxilla and improvement in ANB (Figure 8). The growth treatment response vector (GTRV) analysis was performed using serial lateral cephalograms [10]. The postorthopaedic phase tracing was superimposed onto the 9‐month posttreatment follow‐up cephalogram using stable midsagittal cranial base structures. The functional occlusal plane was constructed using the mesial buccal cusp of the maxillary molar and the incisal tip of the maxillary incisor from the postorthopaedic phase tracing. Perpendicular lines from Points A and B in both radiographs were then constructed to the occlusal plane of the postorthopaedic phase tracing. The horizontal displacement of each point along the same occlusal plane between the two timepoints was then measured. In the present case, Point A demonstrated 3 mm of horizontal advancement, whereas Point B showed 2 mm of forward displacement. Using the formula GTRV  =  horizontal displacement of Point A (3 mm)/horizontal displacement of Point B (2 mm), a ratio of 1.5 was obtained.

**Table 2:** Comparison of cephalometric values at pretreatment (T0), postorthopaedic (T1) and posttreatment (T2).

Variable	Pretreatment (T0)	Postorthopaedic (T1)	Posttreatment (T2)
SNA	80°	81°	83°
SNB	84°	84°	85°
ANB	−4°	−3°	−2°
Wits appraisal	−10 mm	−5 mm	−7 mm
Co‐A	68.5 mm	69 mm	70.5 mm
Co‐Gn	101.5 mm	102.5 mm	105 mm
SnMxP	10°	10°	10°
MMPA	25°	28°	29°
LAFH	53.7%	55%	56.1%
U1MxP	118°	129°	119.5°
L1MnP	76°	69°	84°
Interincisal angle	138°	133°	128°
Lower incisor to APo line	6 mm	2 mm	4.5 mm

**Figure 9:**
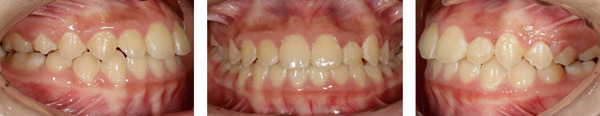
Intraoral findings at 3‐month review appointment after removal of fixed appliance.

**Figure 10:**
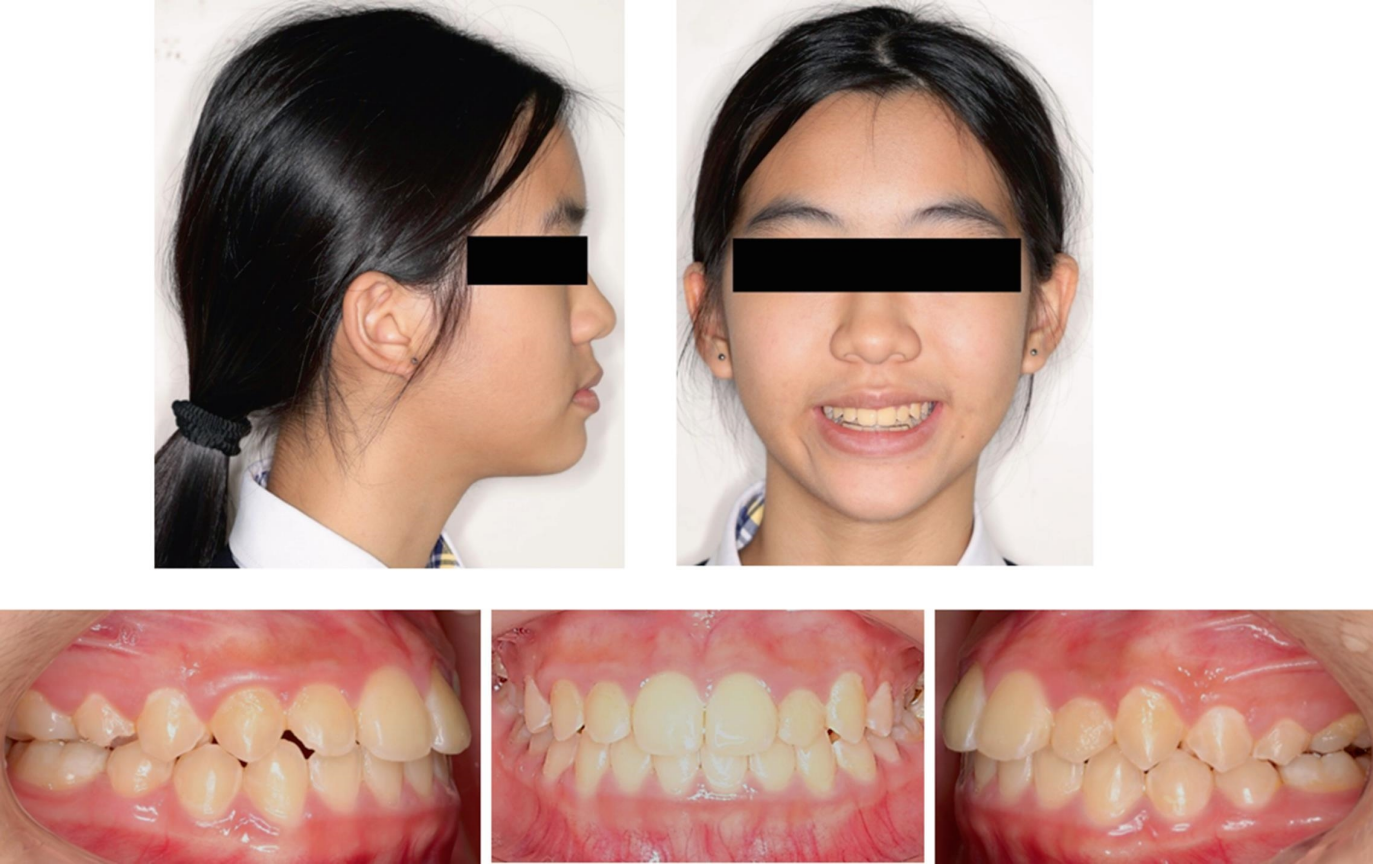
Smile and profile view at 9‐month recall.

The patient tolerated treatment well, with good compliance and no adverse events. At the 3‐month review, the correction of the anterior crossbite and positive overjet was maintained, indicating a stable short‐term result and the patient will be monitored for further growth (Figure 9). At the 9‐month recall, the overjet and overbite remained stable, and the extraoral side profile view showed a Class I skeletal pattern (Figure 10).

## 8. Prognosis

Despite favourable treatment outcomes and stability observed up to 9 months, long‐term stability in skeletal Class III growing patients remains unpredictable. Although overjet, overbite and sagittal relationships were currently maintained, the risk of late mandibular growth and future relapse cannot be excluded. Continued long‐term monitoring into late adolescence is therefore recommended, as some patients may ultimately require orthognathic intervention despite successful early camouflage treatment [4, 7]. The patient and parents are informed of the possible need for orthognathic intervention in the future if such posttreatment changes happen. Nevertheless, the present case demonstrates that meaningful, functional and aesthetic improvement may still be achieved during the growth period.

## 9. Discussion

This case illustrates that early protraction facemask therapy, when initiated according to biological growth stage rather than chronological age, can produce favourable skeletal and dental outcomes in selected patients with skeletal Class III malocclusion. The presence of a positive family history highlights the importance of early intervention and long‐term growth monitoring [11, 12]. Initiation of treatment at cervical vertebral maturation Stage CS2 allowed effective maxillary advancement despite the patient being slightly older than the age range commonly reported in landmark trials [13, 14]. This timing is consistent with evidence reporting greater maxillary advancement when protraction is initiated at CVM Stages CS1 or CS2 [15, 16].

The skeletal and dental changes obtained in this case closely resemble the typical short‐term treatment response of protraction facemask noted in previous studies [13, 14, 17]. In the landmark multicentre randomized controlled trial by Mandall et al. [13], early protraction facemask therapy produced modest but significant forward movement in Point A (mean SNA, +1.4°) and backward movement in Point B (mean SNB, −0.7°), resulting in improvement of ANB (+2.1°) over the short term in the intervention group . Similarly, the present case report demonstrated an increase in SNA from 80° to 81° and improvement in ANB from −4° to −3°, reflecting comparable Class III skeletal correction. Vertically, the mandibular plane angle and lower anterior facial height increased in this case, reflecting both elimination of the functional shift and downward–forward vector of facemask traction. As the patient initially presented with a low‐angle pattern, these vertical changes were favourable in establishing average vertical facial proportions. This increase in mandibular plane angle and lower anterior facial height was in line with the mild vertical opening tendency described by Mandall et al. [13].

The upper incisors were advanced following the orthopaedic phase due to the dentoalveolar effect of the protraction facemask, but their inclination was subsequently corrected during the fixed appliance phase. The lower incisors were initially further retroclined by the orthopaedic phase, but later uprighted to a more aesthetic inclination towards the average during fixed appliance treatment. This was also consistent with the short‐term findings of Mandall et al. [13], who reported a similar tendency for lower incisor retroclination following protraction facemask therapy despite the absence of direct mandibular appliance effects. Favourable facial changes, stable occlusal relationships and a mild residual skeletal discrepancy supported the decision to progress to fixed appliance therapy at an earlier stage. The use of GTRV concept as an indicator of favourable treatment direction is particularly relevant in Asian protraction facemask cohorts [10]. The GTRV is a descriptive cephalometric ratio comparing maxillary to mandibular forward growth using the postorthopaedic and posttreatment serial cephalograms taken 2–4 years apart. It is intended to qualitatively indicate whether a patient′s posttreatment growth pattern is favourable for long‐term camouflage in Class III correction. Although the GTRV in this case was 1.5, which exceeds the range originally reported (0.33–0.88) for successfully treated Class III cases, the elevated value may partly reflect the relatively limited forward displacement of Point B during the observation period, as ratio‐based measurements are sensitive to small denominator changes. Nonetheless, it reflected a relatively favourable maxillary‐to‐mandibular growth relationship in this patient supportive of orthodontic camouflage treatment [10]. The nonexpansion protocol was justified by the absence of a true transverse maxillary deficiency [18].

This case adds to the existing literature by illustrating clinical decision‐making and treatment effectiveness in a borderline skeletal Class III patient with potential for mandibular growth still present. Although conventional protocols favour earlier intervention and often incorporate maxillary expansion, this report demonstrates that a carefully selected patient may still benefit from a nonexpansion facemask approach initiated at a clinically relevant growth stage. The early integration of comprehensive fixed appliance therapy further highlights a streamlined treatment strategy, enabling timely correction of functional shift and improvement in facial aesthetics. Collectively, this case supports a more individualized approach to treatment planning, providing evidence that favourable outcomes may be achievable beyond traditional protocol boundaries in selected cases.

A key limitation of this case was that follow‐up was limited to treatment completion at approximately 4 years, with the absence of long‐term postretention or growth completion data, as the patient remains in the active growth phase. Skeletal Class III growth modification is inherently influenced by the unpredictability of late mandibular growth, particularly in patients with a positive family history of prognathism [19]. Although a favourable treatment response was observed in this case, the lack of long‐term follow‐up means that the stability of the correction beyond adolescence cannot be confirmed. Previous longitudinal studies have demonstrated that relapse may occur due to continued mandibular growth even after successful early intervention [7, 20]. Mandall et al. [20] reported that not all patients maintained positive overjet at long‐term follow‐up at 3 years, highlighting the influence of continued mandibular growth in adolescents. This aligns with the current case, where the long‐term stability remains uncertain. Therefore, continued monitoring until completion of craniofacial growth is essential, and the possibility of future orthodontic camouflage or orthognathic intervention should be acknowledged. Despite that, timely reporting of the two‐phase growth modification treatment outcomes in this case report remains clinically relevant, particularly for informing early intervention strategies in growing Class III patients. Nonetheless, careful case selection, patient compliance and continued follow‐up contributed to a favourable outcome at this stage.

## 10. Conclusion

This case demonstrates that early protraction facemask therapy, when timed appropriately during a favourable growth stage, can result in clinically meaningful and stable correction of skeletal Class III malocclusion. Strategic treatment planning and careful monitoring allowed a successful transition to nonextraction fixed appliance therapy, achieving satisfactory functional and aesthetic outcomes. Continued surveillance remains essential due to the variability of mandibular growth.

## Funding

No funding was received for this manuscript.

## Consent

Written informed consent was obtained from the patient′s parent for publication of clinical details and images. All identifying information has been removed to ensure confidentiality.

## Conflicts of Interest

The authors declare no conflicts of interest.

## Patient Perspective

The patient and her parent expressed satisfaction with the treatment outcome, particularly the improvement in facial appearance and dental alignment. They reported that although appliance wear required commitment, early intervention reduced the perceived need for more invasive treatment later.

## Data Availability

The data that support the findings of this study are available from the corresponding author upon reasonable request.
